# A nomogram to predict 28-day mortality in patients with sepsis combined coronary artery disease: retrospective study based on the MIMIC-III database

**DOI:** 10.3389/fmed.2024.1433809

**Published:** 2024-09-04

**Authors:** Quankuan Gu, Ping Huang, Qiuyue Yang, Xianglin Meng, Mingyan Zhao

**Affiliations:** ^1^Department of Critical Care Medicine, The First Affiliated Hospital of Harbin Medical University, Harbin, Heilongjiang Province, China; ^2^Heilongjiang Provincial Key Laboratory of Critical Care Medicine, Harbin, Heilongjiang Province, China

**Keywords:** 28-day mortality, coronary heart disease, MIMIC-III, nomogram, sepsis

## Abstract

**Object:**

Establish a clinical prognosis model of coronary heart disease (CHD) to predict 28-day mortality in patients with sepsis.

**Method:**

The data were collected retrospectively from septic patients with a previous history of coronary heart disease (CHD) from the Medical Information Mart for Intensive Care (MIMIC)-III database. The included patients were randomly divided into the training cohorts and validation cohorts. The variables were selected using the backward stepwise selection method of Cox regression, and a nomogram was subsequently constructed. The nomogram was compared to the Sequential Organ Failure Assessment (SOFA) model using the C-index, area under the receiver operating characteristic curve (AUC) over time, Net reclassification index (NRI), Integrated discrimination improvement index (IDI), calibration map, and decision curve analysis (DCA).

**Result:**

A total of 800 patients were included in the study. We developed a nomogram based on age, diastolic blood pressure (DBP), pH, lactate, red blood cell distribution width (RDW), anion gap, valvular heart disease, peripheral vascular disease, and acute kidney injury (AKI) stage. The nomogram was evaluated using C-index, AUC, NRI, IDI, calibration plot, and DCA. Our findings revealed that this nomogram outperformed the SOFA score in predicting 28-day mortality in sepsis patients.

## 1 Introduction

Sepsis is caused by an acute infection, which triggers an exaggerated and dysregulated immune response in the host, leading to dysfunction of multiple organs (1). Every year, there are approximately 49 million sepsis patients worldwide (2). Around 30% of patients in the ICU are diagnosed with sepsis (3). The mortality of sepsis can reach up to 40% (4). Patients with sepsis often require different treatment options and may have varying outcomes due to diverse infectious factors, individual variations, and medical history. It is not feasible to evaluate and guide all sepsis patients using a single scoring criterion. The heterogeneity of sepsis patients should receive increasing attention (5, 6).

Coronary heart disease (CHD) is a significant factor that affects the treatment and prognosis of septic patients, and the incidence of fatal CHD in sepsis patients is higher compared to non-septic patients (7). The pathophysiological mechanisms of CHD involve vascular and systemic inflammation, prothrombotic states, vascular stress, altered vascular tone, disrupted hemodynamic homeostasis, and imbalanced metabolism (8). These vascular lesions manifest prominently during the pathological process of sepsis (9, 10). It is often observed in the management of sepsis patients that they have a history of previous CHD. Infection can trigger various cardiovascular events in patients with CHD, including cardiac function deterioration and cardiac arrhythmias (8, 11). Furthermore, a history of CHD can also contribute to increased mortality rates in sepsis patients (12).

When patients with a history of coronary heart disease develop sepsis, it is crucial to establish an accurate prognosis and appropriate treatment plan based on a specialized scoring system. A nomogram is a graphical tool grounded in a statistical prediction model that calculates the probability of a clinical event in a specific patient through multiple indicators (13). However, nomograms for predicting the prognosis of patients with a history of coronary heart disease who have developed sepsis are scarce. In this study, our objective is to develop a nomogram that predicts the outcome for such patients after the onset of sepsis.

## 2 Methods

The Medical Information Mart for Intensive Care (MIMIC)-III database is a significant healthcare resource for critically ill patients. It was developed and is managed by the Massachusetts Institute of Technology (MIT), established in 2003 (14). Our study employed version 1.4 of the MIMIC-III database. This comprehensive database encompasses data from over 58,000 inpatients in the intensive care unit at Beth Israel Deaconess Medical Center between 2001 and 2012 (15). It provides a wealth of real-world data for clinical research, including but not limited to vital signs, medications, laboratory measurements, care provider observations and notes, fluid balance, procedure codes, diagnostic codes, imaging reports, length of hospital stay, and more. All data can be extracted using Structured Query Language (SQL) for further analysis. Participants in this study completed a series of NIH-provided courses and passed the required assessment (certificate number: 62299628).

This retrospective study utilized data from a third-party anonymous, publicly accessible database (MIMIC-III), and received approval from an existing institutional review board. As the patient information in the database was anonymized, informed consent was not necessary for this study. The report of this study adheres to the STROBE guidelines (16).

### 2.1 Study population

**Figure 1:**
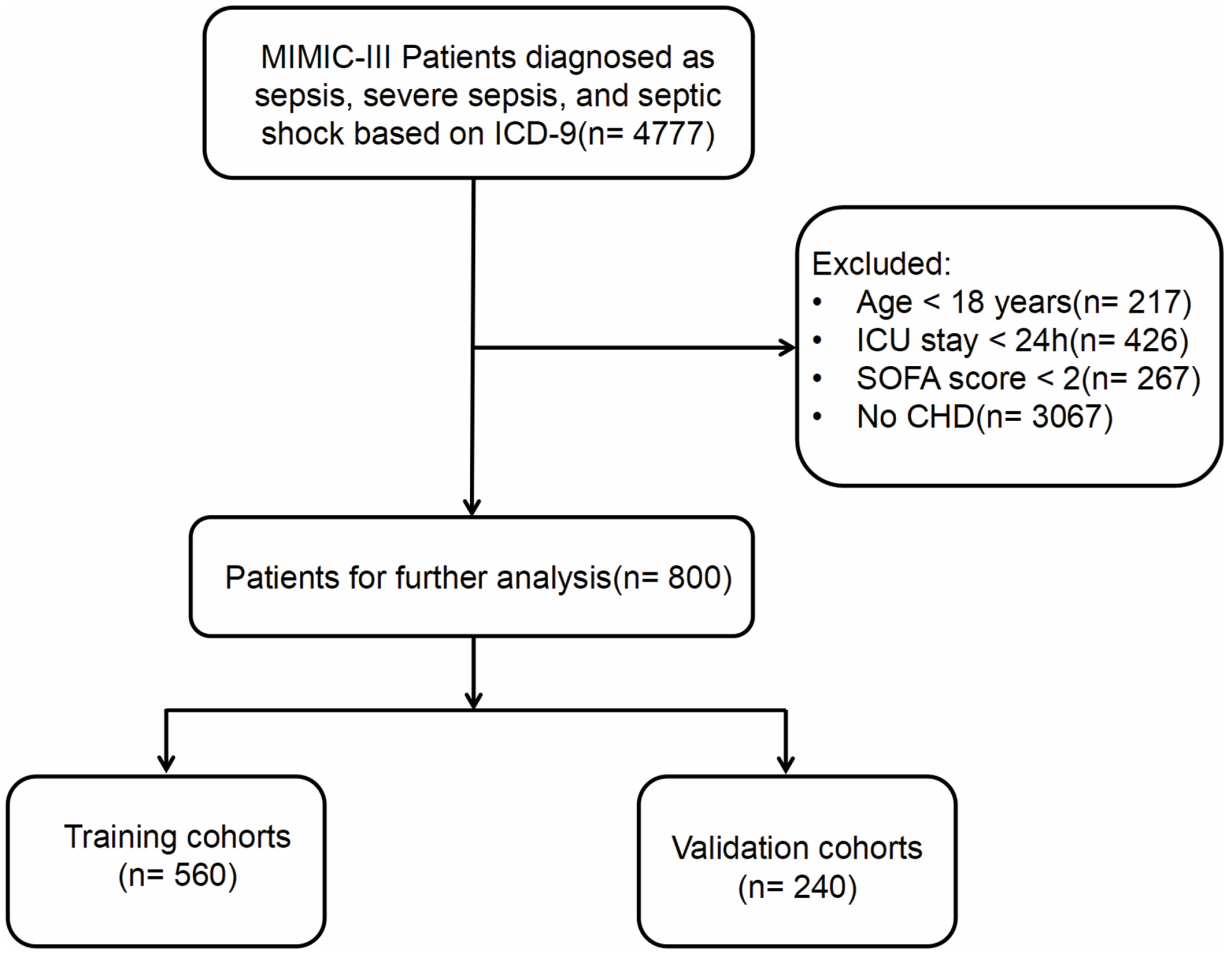
Follow chat of study population selection. MIMIC, Medical Information Mart for Intensive Care; ICD, International Classification of Diseases; SOFA, Sequential Organ Failure Assessment; CHD, Coronary Heart Disease.

The study population was selected based on the Sepsis-3 criteria for diagnosis. Patients diagnosed with sepsis, severe sepsis, and septic shock were extracted from the MIMIC-III database, utilizing the International Classification of Diseases (ICD)-9 code. The exclusion criteria included: (1) patients under the age of 18; (2) patients with an ICU stay of less than 24 h; (3) patients with a SOFA score of less than 2; and (4) patients lacking a prior diagnosis of CHD. For those with multiple ICU admissions, only data from the first ICU admission was extracted. We randomly allocated 70% of the subjects to the training set for this study, reserving the remaining 30% as test data for the validation set. The data extraction process, based on these inclusion criteria, is represented in Figure 1.

### 2.2 Research method

We utilized SQL to extract the following information from the MIMIC-III database: age, sex, weight, race, admission type, initial care unit, SOFA score, and Acute Physiology Score III (APSIII). Additionally, we obtained data on interventions such as ventilators, continuous Renal Replacement Therapy (CRRT), and vasoactive drugs; complications including congestive heart failure, arrhythmia, valvular heart disease, peripheral vascular disease, renal failure, liver disease, hypertension, diabetes, obesity, and AKI, among others; laboratory test results such as white blood cell count (WBC), neutrophil percentage, red blood cell distribution width(RDW), hematocrit, sodium, potassium, albumin, lactate, and blood pH; and vital signs, including temperature, heart rate, respiratory rate, blood pressure, and SpO_2_. All this aforementioned data corresponds to the 24 h prior to ICU admission. The primary outcome measure was the patients’ 28-day mortality, which was obtained from the patient hospitalization data in the MIMIC-III database.

### 2.3 Statistical analysis

Continuous variables that met the normal distribution were represented by mean ± SD values, while non-normally distributed continuous variables were represented by the median and quartiles [M (Q1, Q3)]. Categorical variables were presented in percentages. Stepwise regression and Cox regression were utilized in the selection of variables for the model (17), choosing the method with higher sensitivity. The predictive model was constructed using logistic regression, to estimate the 28-day mortality among septic patients with prior coronary heart disease.

To evaluate the discriminative ability of the model, we employed Harrell’s concordance index (C-index), which measures the model’s prediction accuracy and enables comparison with the existing SOFA indicator (represented by the area under the curve, AUC). The AUC scale extends from 0 to 1, where 1 signifies complete agreement, and 0.5 suggests that the model’s performance is no better than chance. Larger AUC values denote more accurate prognostic stratification. Calibration curves were constructed using bootstrapping with 500 resamplings to assess the agreement between the predicted survival probability by the model and the observed adverse outcomes, thereby validating the clinical applicability of the model based on decision curve analysis (DCA).

All statistical analyses were conducted using the R software version 4.2.2 (R Foundation for Statistical Computing, Vienna, Austria). All tests were two-sided, and a *p*-value less than 0.05 was considered statistically significant.

## 3 Results

### 3.1 Basic characteristics of the study subjects

A total of 4,777 patients were identified through the screening criteria based on the ICD codes. After applying the exclusion criteria, 800 patients were ultimately included in the final dataset. These patients were randomly assigned to Training cohorts (*n* = 560) and Validation cohorts (*n* = 240). Patient baseline characteristics are presented in Table 1. The Training cohort consisted of 349 males (62.3%) and 211 females (37.7%), with a median age of 70 years (IQR = 56–81 years). The Validation cohort included 158 males (65.8%) and 82 females (34.2%), with a median age of 68 years (IQR = 57–80 years). The majority of patients in both cohorts were white(>70%), and most admissions were emergencies (>95%). The most common initial care unit was the MICU (68.4% vs. 67.9%). Over 60% of the patients had health insurance. The median body temperature for both groups was 37.0°C (IQR = 36.0–37.0°C), and the median heart rate was 92 bpm (IQR = 79–104.25 bpm) and 91 bpm (IQR = 80–104 bpm). The median respiratory rate was 21 bpm (IQR = 17.75–24 bpm vs. 18–24 bpm). The median systolic blood pressure was 107 mmHg (IQR = 101–116 vs. 100–116 mmHg). Both groups had median diastolic blood pressures of 56 mmHg (IQR = 50–63 and 50–61.25 mmHg). The median SOFA score was 7.0 in both groups (IQR = 4–10 vs. 4–9). Median APSIII scores were 60 (IQR = 46–75) and 58.5(IQR = 41.75–77). In these patients, the majority had a pH less than 7.35 (45.7% vs. 45.4%). Over 60% of patients had lactate levels ranging between 0.5 and 1.6 mmol/L. Abnormalities in WBC count were observed in 71.3 and 67.1% of patients. RDW was abnormal in 69.3 and 64.6% of the Training and Validation cohorts, respectively. Of these patients, 57 and 51.2% received mechanical ventilation, 21 and 20% used vasopressors, and 12 and 8.6% underwent CRRT. A majority of patients had comorbidities such as congestive heart failure (64 and 59.6%), hypertension (68 and 70.4%), diabetes mellitus (33 and 30.4%), and renal failure (40 and 44.2%). Over 80% of the patients in both groups developed AKI. There were no statistical difference in basic characteristics in two groups except PaCO_2_ (*p* = 0.022). Missing data values obtained for all 800 patients were below 20%. If the missing data followed a normal distribution, the mean was used to fill in the missing data. If it did not, the median was used. The all-cause mortality rate in all patients was 66.8% (*n* = 535). The mortality rates in the Training and Validation cohorts were 65.9% (*n* = 369) and 69.2% (*n* = 166), respectively.

**Table 1:** Baseline characteristics of included participants.

	All (*n* = 800)	Training cohorts (*n* = 560)	Validation cohorts (*n* = 240)	Statistics	P
Age, years, M (Q1, Q3)	69.00 (57.00, 81.00)	70.00 (56.00, 81.00)	68.00 (57.00, 80.00)	Z = 0.58	0.507
Gender, male(%)	507 (63.4)	349 (62.3)	158 (65.8)	*χ^2^* = 0.893	0.345
Weight, Kg, M (Q1, Q3)	77.00 (64.00, 91.00)	77.5 (65.00, 91.00)	75.00 (63.75, 91.00)	Z = 0.884	0.612
Race, n(%)				*χ^2^* = 3.392	0.335
White	600 (75)	419 (74.8)	181 (75.4)		
Black	74 (9.3)	47 (8.4)	27 (11.3)		
Asian	18 (2.2)	12 (2.2)	6 (2.5)		
Other	108 (13.5)	82 (14.6)	26 (10.8)		
Admission type, n(%)				*χ^2^* = 0.151	0.927
EMERGENCY	762 (95.3)	533 (95.2)	229 (95.4)		
ELECTIVE	27 (3.2)	18 (3.2)	8 (3.3)		
URGENT	12.(1.5)	9 (1.6)	3 (1.3)		
First careunit, n(%)				*χ^2^* = 5.26	0.154
MICU	546 (68.3)	383 (68.4)	163 (67.9)		
SICU	93 (11.6)	65 (11.6)	28 (11.7)		
CCU	81 (10.1)	63 (11.2)	18 (7.5)		
Other	80 (10)	49 (8.8)	31 (12.9)		
Insurance, n(%)				*χ^2^* = 4.474	0.215
Medicare	478 (59.8)	347 (62.0)	131 (54.6)		
Private	222 (27.8)	145 (25.8)	77 (32.1)		
Medicaid	74 (9.2)	49 (8.8)	25 (10.4)		
Other	26 (3.2)	19 (3.4)	7 (2.9)		
Vital signs					
Temperature, °C, M (Q1, Q3)	37.00 (36.00, 37.00)	37.00 (36.00, 37.00)	37.00 (36.00, 37.00)	Z = 1.471	0.1
Heart rate, bpm, M (Q1, Q3)	92.00 (79.00, 104.00)	92.00 (79.00, 104.25)	91.00 (80.00, 104.00)	Z = 0.429	0.668
Respiratory rate, bpm, M (Q1, Q3)	21.00 (18.00, 24.00)	21.00 (17.75, 24.00)	21.00 (18.00, 24.00)	Z = 0.435	0.664
SBP, mmHg, M (Q1, Q3)	107.00 (101.00, 116.00)	107.00 (101.00, 116.00)	107.00 (100.00, 116.00)	Z = 0.023	0.982
DBP, mmHg, M (Q1, Q3)	56.00 (50.00, 62.00)	56.00 (50.00, 63.00)	55.00 (50.00, 61.25)	Z = 1.123	0.262
MAP, mmHg, M (Q1, Q3)	71.00 (65.75, 77.00)	71.00 (66.00, 77.00)	70.00 (65.00, 77.00)	Z = 1.046	0.296
SpO_2_, %, M (Q1, Q3)	97.00 (96.00, 98.00)	97.00 (96.00, 98.00)	97.00 (96.00, 98.00)	Z = 0.058	0.954
SOFA, score, M (Q1, Q3)	7.00 (4.00, 9.00)	7.00 (4.00, 10.00)	7.00 (4.00, 9.00)	Z = 1.006	0.315
APSIII, score, M (Q1, Q3)	60.00 (45.00, 76.00)	60.00 (46.00, 75.00)	58.50 (41.75, 77.00)	Z = 1.343	0.18
Blood gas analysis					
pH, n(%)				*χ^2^* = 1.240	0.536
7.35–7.45	321 (40.1)	229 (40.9)	92 (38.3)		
< 7.35	365 (45.6)	256 (45.7)	109 (45.4)		
> 7.45	114 (14.3)	75 (13.4)	39 (16.3)		
PaO_2_, mmHg, n(%)				*χ^2^* = 2.183	0.336
80–100	131 (16.4)	89 (15.9)	42 (17.5)		
< 80	246 (30.8)	181 (32.3)	65 (27.1)		
> 100	423 (52.8)	290 (51.8)	133 (55.4)		
PCO_2_, mmHg, n(%)				χ^2^ = 7.645	0.022
35–45	334 (41.8)	232 (41.4)	102 (42.5)		
< 35	201 (25.1)	128 (22.9)	73 (30.4)		
> 45	265 (33.1)	200 (35.7)	65 (27.1)		
Lactate, mmol/L, n(%)				*χ^2^* = 1.109	0.292
0.5–1.6	502 (62.8)	358 (63.9)	144 (60.0)		
< 0.5 OR > 1.6	298 (37.2)	202 (36.1)	96 (40.0)		
Glucose, mg/dL, M (Q1, Q3)	139.00 (113.00, 170.20)	139.00 (113.00, 170.00)	137.50 (113.80, 172.00)	Z = 0.524	0.601
Laboratory test					
WBC, K/μL, n(%)				*χ^2^* = 1.389	0.239
3.5–9.5	240 (30)	161 (28.7)	79 (32.9)		
< 3.5 OR > 9.5	560 (70)	399 (71.3)	161 (67.1)		
Neutrophil, %, n(%)				*χ^2^* = 0.019	0.89
50–75	151 (18.9)	105 (18.8)	46 (19.2)		
< 50 OR > 75	649 (81.1)	455 (81.2)	194 (80.8)		
Hemoglobin, g/dL, n(%)				*χ^2^* = 0.0722	0.788
12–16	162 (20.3)	112 (20.0)	50 (20.8)		
< 12 OR > 16	638 (79.7)	448 (80.0)	190 (79.2)		
RDW, %, n(%)				*χ^2^* = 1.704	0.192
11.5–14.5	257 (32.1)	172 (30.7)	85 (35.4)		
< 11.5 OR > 14.5	543 (67.9)	388 (69.3)	155 (64.6)		
Hematocrit, n(%)				*χ^2^* = 0.1614	0.688
35–45	186 (23.3)	128 (22.9)	58 (24.2)		
< 35 OR > 45	614 (76.7)	432 (77.1)	182 (75.8)		
Platelet, K/μL, n(%)				*χ^2^* = 0.097	0.755
100–300	537 (67.1)	374 (66.8)	163 (67.9)		
< 100 OR > 300	263 (32.9)	186 (33.2)	77 (32.1)		
PT, sec, n(%)				*χ^2^* = 0.69	0.406
9.8–12.1	63 (7.9)	47 (8.4)	16 (6.7)		
< 9.8 OR > 12.1	737 (92.1)	513 (91.6)	224 (93.3)		
PTT, sec, n(%)				*χ^2^* = 0.379	0.828
25–31.3	296 (37.0)	211 (37.7)	85 (35.4)		
< 25	93 (11.6)	64 (11.4)	29 (12.1)		
> 31.3	411 (51.4)	285 (50.9)	126 (52.5)		
INR, ratio, n(%)				*χ^2^* = 0.526	0.468
0.8–1.2	318 (39.8)	218 (38.9)	100 (41.7)		
< 0.8 OR > 1.2	482 (60.2)	342 (61.1)	140 (58.3)		
Albumin, g/dL, n(%)				*χ^2^* = 1.131	0.288
3.5–5.5	184 (23)	123 (22.0)	61 (25.4)		
< 3.5 OR > 5.5	616 (77)	437 (78.0)	179 (74.6)		
ALT, U/L, n(%)				*χ^2^* = 0.188	0.665
5–40	509 (63.6)	359 (64.1)	150 (62.5)		
< 5 OR > 40	291 (36.4)	201 (35.9)	90 (37.5)		
AST, U/L, n(%)				*χ^2^* = 0.004	0.951
8–40	418 (52.3)	293 (52.3)	125 (52.1)		
< 8 OR > 40	382 (47.7)	267 (47.7)	115 (47.9)		
Total bilirubin, mg/dL, n(%)				*χ^2^* = 2.216	0.137
0.3–1.3	543 (67.9)	371 (66.2)	172 (71.7)		
< 0.3 OR > 1.3	257 (32.1)	189 (33.8)	68 (28.3)		
BUN, mg/dL, n(%)				*χ^2^* = 3.743	0.053
9–20	163 (20.4)	104 (18.6)	59 (24.6)		
< 9 OR > 20	637 (79.6)	456 (81.4)	181 (75.4)		
Creatinine, mg/dL, n(%)				*χ^2^* = 0.066	0.797
0.6–1.2	225 (28.1)	156 (27.9)	69 (28.7)		
< 0.6 OR > 1.2	575 (71.9)	404 (72.1)	171 (71.3)		
LDH, U/L, n(%)				*χ^2^* = 0.012	0.913
100–300	471 (58.9)	329 (58.7)	142 (59.2)		
< 100 OR > 300	329 (41.1)	231 (41.3)	98 (40.8)		
K^+^, mmol/L, n(%)				*χ^2^* = 2.694	0.26
3.5–5.5	581 (72.6)	412 (73.6)	169 (70.4)		
< 3.5	214 (26.8)	146 (26.1)	68 (28.3)		
> 5.5	5 (0.6)	2 (0.3)	3 (1.3)		
Na^+^, mmol/L, n(%)				*χ^2^* = 0.046	0.977
135–145	506 (63.3)	353 (63)	153 (63.7)		
< 135	261 (32.6)	184 (33)	77 (32.1)		
> 145	33 (4.1)	23 (4)	10 (4.2)		
Anion gap, mmol/L, n(%)				*χ^2^* = 0.313	0.855
8–16	628 (78.5)	437 (78.0)	191 (79.6)		
< 8 or > 16	172 (21.5)	123 (22.0)	49 (20.4)		
Intervention					
Ventilation, n(%)	444 (55.5)	321 (57)	123 (51.2)	*χ^2^* = 2.507	0.113
Vasopressor, n(%)	168 (21)	120 (21)	48 (20.0)	*χ^2^* = 0.207	0.649
CRRT, n(%)	88 (11)	67 (12)	21 (8.6)	*χ^2^* = 1.773	0.183
Aspirin, n(%)	411 (51.3)	287 (51.2)	124 (51.7)	*χ^2^* = 0.012	0.914
Heparin, n(%)	453 (56.6)	317 (56.6)	136 (56.7)	*χ^2^* = 0.0002	0.987
Statin, n(%)	296 (37)	210 (37.5)	88 (36.7)	*χ^2^* = 0.582	0.483
Complication					
Congestive heart failure, n(%)	500 (62.5)	357 (64)	143 (59.6)	*χ^2^* = 1.244	0.265
Cardiac arrhythmias, n(%)	470 (58.8)	333 (59)	137 (57.1)	*χ^2^* = 0.393	0.537
Valvular heart disease, n(%)	156 (19.5)	119 (21)	37 (15.4)	*χ^2^* = 3.642	0.056
Pulmonary circulation disease, n(%)	73 (9.1)	50 (9)	23 (9.6)	*χ^2^* = 0.087	0.768
Peripheral vascular disease, n(%)	161 (20.1)	124 (22)	37 (15.4)	*χ^2^* = 4.728	0.03
Hypertension, n(%)	253 (31.6)	378 (68)	169 (70.4)	*χ^2^* = 0.661	0.416
Chronic pulmonary, n(%)	239 (29.9)	171 (31)	68 (28.3)	*χ^2^* = 0.389	0.533
Diabetes, n(%)	258 (32.3)	185 (33)	73 (30.4)	*χ^2^* = 0.527	0.468
Renal failure, n(%)	330 (41.3)	224 (40)	106 (44.2)	*χ^2^* = 1.204	0.273
Liver disease, n(%)	110 (13.8)	81 (14)	29 (12.1)	*χ^2^* = 0.803	0.37
Peptic ulcer, n(%)	9 (1.1)	7 (1)	2 (0.8)	*χ^2^* = 0.262	0.609
Obesity, n(%)	58 (7.3)	41 (7.3)	17 (7.1)	*χ^2^* = 0.014	0.905
Weight loss, n(%)	49 (6.1)	34 (6.1)	15 (6.2)	*χ^2^* = 0.009	0.923
Alcohol abuse, n(%)	31 (3.9)	23 (4.1)	8 (3.3)	*χ^2^* = 0.27	0.603
AKI stage, n(%)				*χ^2^* = 3.207	0.361
NO	86 (10.8)	62 (11.1)	24 (10.0)		
stage I	176 (22)	115 (20.5)	61 (25.4)		
stage II	239 (29.9)	175 (31.3)	64 (26.7)		
stage III	299 (37.3)	208 (37.1)	91 (37.9)		
LOS, days, M (Q1, Q3)	10.38 (5.92, 19.75)	10.08 (5.83, 19.375)	11.00 (6.29, 20.21)	Z = 1.042	0.057
Status, n(%)				*χ^2^* = 0.812	0.367
Survival	265 (33.1)	191 (34.1)	74 (30.8)		
Death	535 (66.9)	369 (65.9)	166 (69.2)		

AKI, Acute kidney injury; ALT, Alanine aminotransferase; APSIII, Acute Physiology Score III; AST, Aspartate aminotransferase; BUN, Blood urea nitrogen; CCU, Cardiac care unit; CRRT, Continuous Renal Replacement Therapy; DBP, Diastolic blood pressure; INR, International normalized ratio; LDH, Lactate dehydrogenase; LOS, Lengths of stay; M, Median; MAP, Mean arterial pressure; MICU, Medical critical care unit; PT, Prothrombin time; PTT, Partial thromboplastin time; Q1, 1st quartile; Q3, 3st quartile; RDW, Red blood cell volume distribution width; SBP, Systolic blood pressure; SICU, Surgical critical care unit; SOFA, Sequential Organ Failure Assessment; SpO_2_, peripheral oxygen saturation; t, t test; WBC, White blood cell; Z, Wilcoxon rank sum test; χ^2^, Chi-square test.

**Figure 2:**
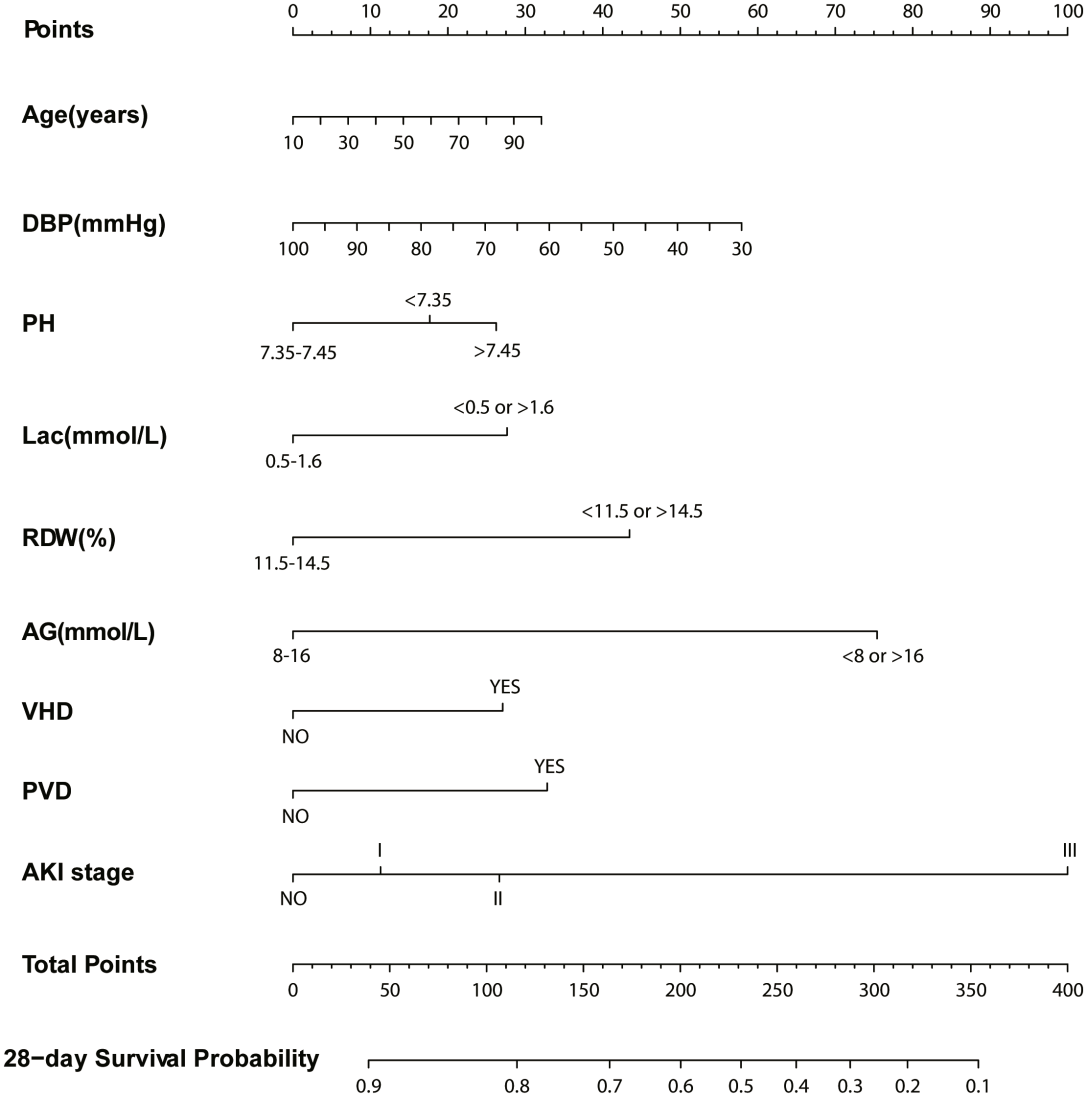
Nomogram for predicting 28-day survival. Left column shows the points bar (top) and nine parameters, each to be scored with a vertical line to the points bar, according to the different parameter values. The sum of the points is calculated (total points range, 0–400), and a vertical line is drawn from the total points bar to the 28-days survival probability below, to obtain survival probability of the patient. DBP, Diastolic blood pressure; Lac, Lactate; RDW, Red blood cell volume distribution width; AG, Anion gap; VHD, Valvular heart disease; PVD, Peripheral vascular disease; AKI, Acute kidney injury.

Based on the Cox regression and the stepwise regression method, the following variables were included in the preliminary model: age (hazard ratio [HR]: 1.01, 95% confidence interval [CI]: 1–1.02, *p* = 0.049); diastolic blood pressure (DBP; HR: 0.97, CI: 0.94–0.992, *p* = 0.034); pH values (pH < 7.35, HR: 1.4, CI 1.05–1.88, *p* = 0.022, pH > 7.45: HR: 1.55, CI: 1.05–2.29, *p* = 0.049); lactic acid (HR: 1.45, CI: 1.11–1.91, *p* = 0.007); RDW (HR: 1.51, CI: 1.13–2.01, *p* = 0.005); anion gap (HR: 4.15, CI: 1.36–11.09, *p* < 0.001); valvular heart disease (HR: 1.54, CI: 1.12–2.1, *p* = 0.007); peripheral vascular disease (HR: 1.65, CI: 1.22–2.23, *p* = 0.001); and AKI stage (stage I: HR: 1.34, CI: 0.81–2.22, *p* = 0.256, stage II: HR: 1.49, CI: 0.91–2.43, *p* = 0.11, stage III: HR: 2.88, CI: 1.78–4.67, *p* < 0.001). A nomogram was established using these nine selected variables. The nomogram functions by assigning a score to each variable on a corresponding scale. The scores of all variables are then summed to obtain a total score, which is used to estimate the probability of sepsis occurrence by drawing a vertical line on the main axis (Figure 2). HRs for other indicators, 95% CI, and statistical measures are presented in Table 2.

**Table 2:** Selected variables analyzed by multivariable Cox regression in the training cohort.

	HR	95%CI(Lower)	95%CI(Upper)	*p*
Age, years	1.01	1	1.02	0.049*
Sex				
Male	Reference			
Female	0.8	0.61	1.05	0.106
Weight, Kg	1.0	0.99	1.01	0.321
Temperature, °C	1.01	0.85	1.21	0.875
Heart rate, min^−1^	1.0	0.99	1.02	0.487
Respiratory rate, min^−1^	1.01	0.99	1.04	0.122
SBP, mmHg	0.99	0.98	1.01	0.786
DBP, mmHg	0.97	0.94	0.99	0.034*
MAP, mmHg	1.03	0.99	1.08	0.091
SpO_2_, %	1.0	0.95	1.07	0.879
SOFA, score	0.99	0.95	1.05	0.919
APSIII, score	0.99	0.99	1.01	0.924
pH				
7.35–7.45	Reference			
< 7.35	1.4	1.05	1.88	0.022*
> 7.45	1.55	1.05	2.29	0.029*
PaO_2_, mmHg				
80–100	Reference			
< 80	0.93	0.65	1.34	0.693
> 100	0.71	0.50	0.99	0.049
Lactate, mmol/L				
0.5–1.6	Reference			
< 0.5 OR > 1.6	1.45	1.11	1.91	0.007**
Glucose, mg/dL	1.0	0.99	1.0	0.39
WBC, K/μl				
3.5–9.5	Reference			
< 3.5 OR > 9.5	0.89	0.68	1.18	0.425
Hemoglobin, g/dL				
12–16	Reference			
< 12 OR > 16	1.19	0.72	1.94	0.5
RDW, %				
11.5–14.5	Reference			
< 11.5 OR > 14.5	1.51	1.13	2.01	0.005**
Hematocrit				
35–45	Reference			
< 35 OR > 45	0.84	0.53	1.33	0.448
Platelet, K/μL				
100–300	Reference			
< 100 OR > 300	0.98	0.75	1.28	0.884
PT, sec				
9.8–12.1	Reference			
< 9.8 OR > 12.1	1.29	0.77	2.20	0.33
PTT, sec				
25–31.3	Reference			
< 25	1.47	0.97	2.24	0.072
> 31.3	0.94	0.71	1.23	0.629
INR, ratio				
0.8–1.2	Reference			
< 0.8 OR > 1.2	1.23	0.89	1.68	0.204
Albumin, g/dL				
3.5–5.5	Reference			
< 3.5 OR > 5.5	1.19	0.86	1.63	0.29
ALT, U/L				
5–40	Reference			
< 5 OR > 40	0.75	0.54	1.05	0.091
AST, U/L				
8–40	Reference			
< 8 OR > 40	0.88	0.64	1.20	0.415
Total bilirubin, mg/dL				
0.3–1.3	Reference			
< 0.3 OR > 1.3	0.96	0.73	1.26	0.764
BUN, mg/dL				
9–20	Reference			
< 9 OR > 20	1.25	0.85	1.85	0.261
Creatinine, mg/dL				
0.6–1.2	Reference			
< 0.6 OR > 1.2	0.75	0.53	1.05	0.089
LDH, U/L				
100–300	Reference			
< 100 OR > 300	1.15	0.88	1.50	0.305
K^+^, mmol/L				
3.5–5.5	Reference			
< 3.5	0.9	0.67	1.21	0.492
> 5.5	1.34	0.26	6.84	0.727
Na^+^, mmol/L				
135–145	Reference			
< 135	1.19	0.86	1.63	0.293
> 145	0.98	0.49	1.93	0.951
Anion gap, mmol/L				
8–16	Reference			
< 8 or > 16	4.15	1.36	11.09	<0.001***
Congestive heart failure				
YES	Reference			
NO	1.03	0.78	1.34	0.848
Cardiac arrhythmias				
YES	Reference			
NO	1.15	0.89	1.48	0.299
Valvular heart disease				
YES	Reference			
NO	1.54	1.12	2.1	0.007**
Pulmonary circulation disease				
YES	Reference			
NO	0.91	0.57	1.47	0.704
Peripheral vascular disease				
YES	Reference			
NO	1.65	1.22	2.23	0.001***
Hypertension				
YES	Reference			
NO	0.84	0.65	1.09	0.198
Chronic pulmonary				
YES	Reference			
NO	1.05	0.78	1.41	0.761
Diabetes				
YES	Reference			
NO	0.97	0.73	1.29	0.856
Renal failure				
YES	Reference			
NO	1.0	0.75	1.34	0.984
Liver disease				
YES	Reference			
NO	1.05	0.73	1.51	0.789
Peptic ulcer				
YES	Reference			
NO	0.72	0.23	2.25	0.57
Obesity				
YES	Reference			
NO	0.61	0.37	1.01	0.055
Weight loss				
YES	Reference			
NO	1.05	0.64	1.72	0.836
Alcohol abuse				
YES	Reference			
NO	1.19	0.61	2.33	0.617
AKI stage, n(%)				
NO	Reference			
stage I	1.34	0.81	2.22	0.256
stage II	1.49	0.91	2.43	0.11
stage III	2.88	1.78	4.67	<0.001****

AKI, Acute kidney injury; ALT, Alanine aminotransferase; APSIII, Acute Physiology Score III; AST, Aspartate aminotransferase; BUN, Blood urea nitrogen; DBP, Diastolic blood pressure; INR, International normalized ratio; LDH, Lactate dehydrogenase; LOS, Lengths of stay; MAP, Mean arterial pressure; PT, Prothrombin time; PTT, Partial thromboplastin time; RDW, Red blood cell volume distribution width; SBP, Systolic blood pressure; SICU, Surgical intensive care unit; SOFA, Sequential Organ Failure Assessment; SpO_2_, peripheral oxygen saturation; WBC, White blood cell, *<0.05; **<0.01; ***<0.005; ****<0.001.

### 3.2 Discriminative ability of the nomogram

**Figure 3:**
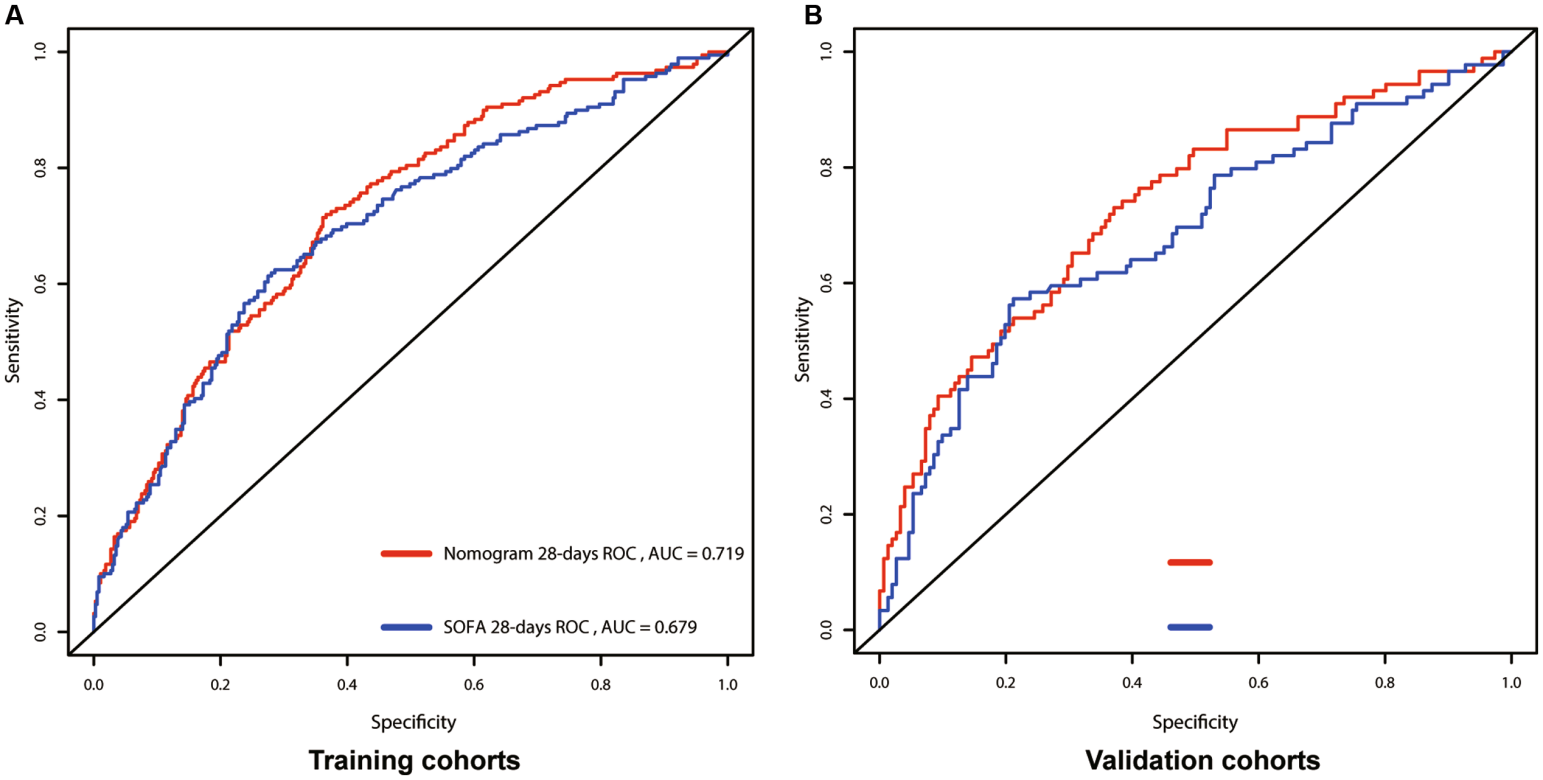
Receiver operating characteristic (ROC) curves for the nomogram and SOFA model, showing AUCs for: 28-days survival. **(A)** Training cohort; **(B)** Validation cohort; SOFA, Sequential Organ Failure Assessment; AUC, area under the curve.

**Figure 4:**
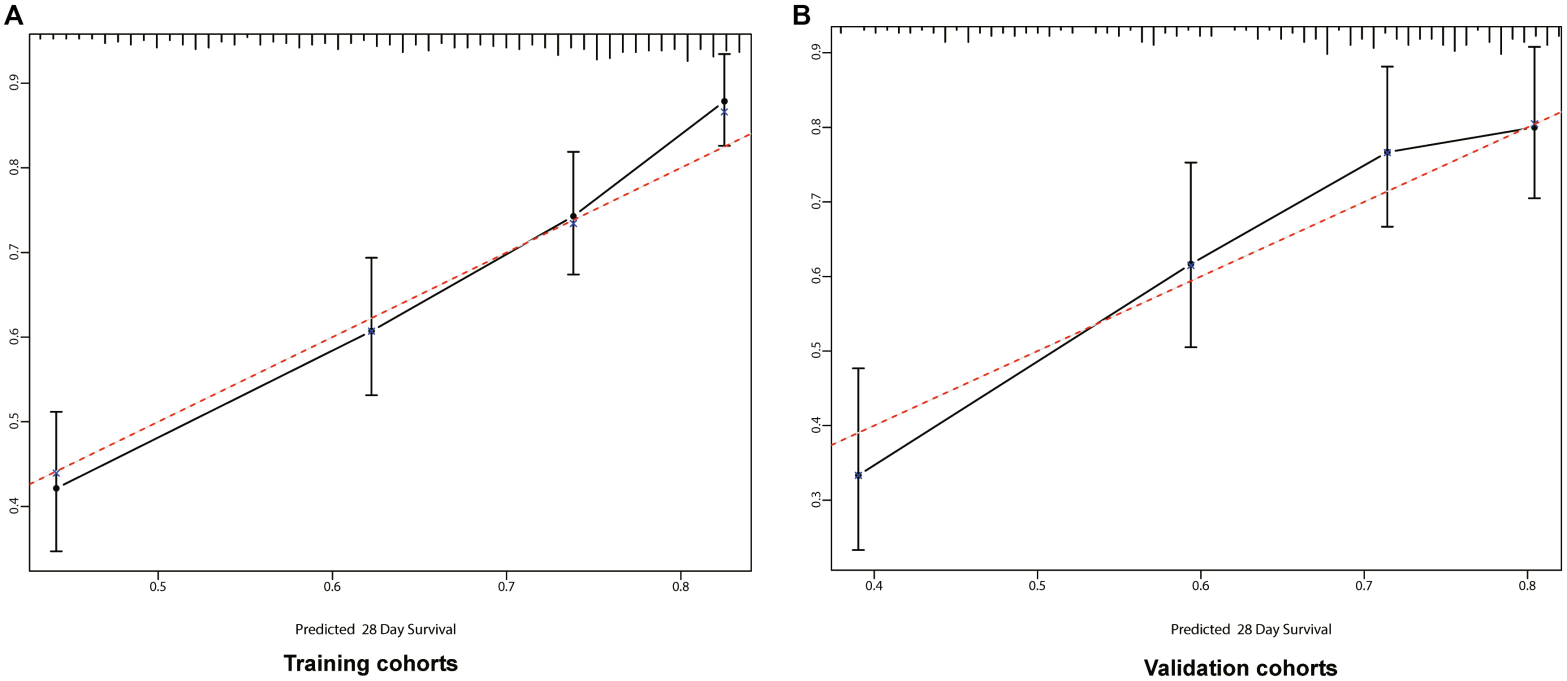
Calibration curves for 28-days survival. The abscissa (x-axis) is the predicted survival rate and the ordinate (y-axis) is the actual survival rate. The red dotted line is the reference line (predicted value equals the actual value), the solid black line is the curve fitting line, and the error bars represent 95% confidence intervals. The calibration curves depict the agreement between predicted probabilities and observed outcomes. **(A)** Training cohort; **(B)** Validation cohort.

We utilized C-index, AUC, NRI, and IDI metrics to evaluate the nomogram’s performance. The C-index for the nomogram of the Training cohort was higher than that of the SOFA score (0.667 vs. 0.661). Similarly, the C-index of the Validation cohort’s nomogram also surpassed the SOFA score (0.661 vs. 0.659). These findings were further confirmed by the AUC plots. The AUC for the Training and Validation cohorts were 0.719 (SOFA: 0.679) and 0.724 (SOFA: 0.684; Figure 3), respectively. The median NRI value was 0.159 (95% CI: 0.048–0.364) in the Training cohort and 0.269 (95% CI, 0.048–0.613) in the Validation cohort. The IDI was 0.121 (*p* < 0.001) for the Training cohort, and 0.103 (*p* < 0.001) for the Validation cohort (Figure 4).

### 3.3 Nomogram calibration

We incorporated variables such as age, DBP, pH, lactate levels, RDW, anion gap, valvular heart disease, peripheral vascular disease, and AKI stage into a preliminary model, leading to the establishment of a nomogram for predicting the prognosis of sepsis patients. The congruence between the calibration curve and the standard curve in the training and validation cohorts’ calibration plots indicates that the 28-day survival prediction aligns with the observed outcomes (Figure 4).

**Figure 5:**
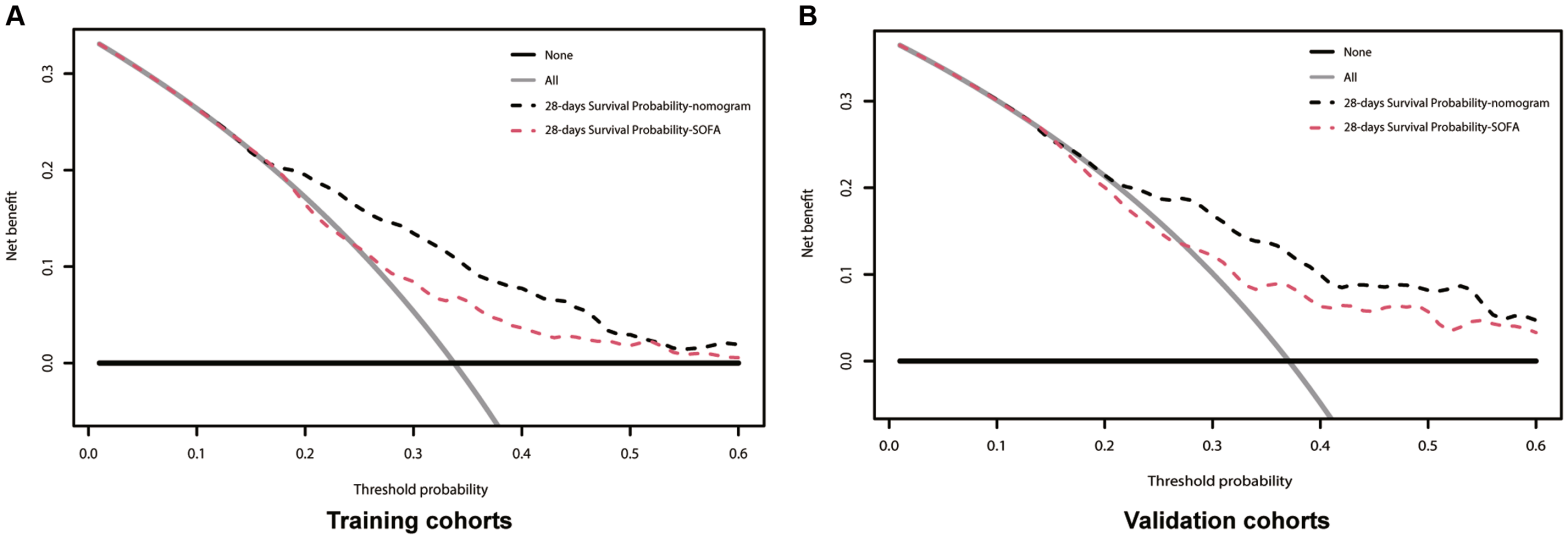
Decision-curve analysis of the nomogram for 28-days survival. In all figures the black line is above the red line, showing that the area under the curve is larger for the new nomogram model than for the SOFA model. **(A)** Training cohort; **(B)** Validation cohort; SOFA, Sequential Organ Failure Assessment; Black dotted line, nomogram model; Red dotted line, SOFA model.

The above results show that our clinical precautionary model is superior to the SOFA score in terms of sensitivity and specificity. In order to verify whether this clinical pre-curative model will be beneficial in clinical practice, we used a DCA curve. In both the Training and Validation cohorts, the nomogram (black line) is above the SOFA score (red line). The net benefit of the nomogram outperformed the SOFA score at any given predicted probability (Figure 5), suggesting that the nomogram plays a significant role in predicting 28-day mortality.

## 4 Discussion

A search of the MIMIC-III database identified 4,777 patients who met the diagnostic criteria for sepsis, of which 800 ultimately met the inclusion criteria. Based on the data from these 800 patients, we found that age, DBP, pH, lactic acid levels, RDW, anion gap, valvular heart disease, peripheral vascular disease, and AKI grade were all correlated with the 28-day mortality of septic patients with a history of coronary heart disease. To optimize the 28-day mortality of such patients, we developed a nomogram, which clinical practitioners can use in the future to better analyze the prognosis of patients with a history of coronary heart disease.

Among the 800 patients included in the study, the all-cause mortality rate was 66.9% (*n* = 535), which is higher than that of sepsis alone (40%) (4). This suggests that septic patients with a history of coronary heart disease have a higher risk of mortality. Moreover, for the majority of patients (*n* > 70%), several indicators including pH, PaO2, WBC, hemoglobin, RDW, HCT, PT, PTT, albumin, BUN, and creatinine were within abnormal ranges. Over 80% of the patients also had concurrent AKI. A higher number of patients required vasopressor support, and a significant number underwent CRRT.

Age is an independent risk factor for most diseases, and our study aligns with this conclusion. For most patients, the objective of blood pressure management is to maintain it within a suitable range; extremes in either direction can be detrimental. Intriguingly, our study revealed that a moderately elevated DBP is advantageous for the prognosis of septic patients with CHD. A pediatric sepsis study showed that survivors had notably higher DBP than non-survivors, and low DBP could serve as an independent risk factor for 28-day survival following a multivariate factor analysis (18). This could be due to the potential for higher DBP levels to mitigate tissue hypoperfusion and decrease the likelihood of septic shock, providing a fresh perspective for sepsis treatment. Our findings also suggest that RDW can be used as a prognostic factor in septic patients with CHD. Other studies have identified RDW as a predictor of all-cause mortality in sepsis patients (19). Furthermore, an abnormal anion gap was associated with a decreased 28-day mortality. When Xu Sun et al. analyzed data from critically ill surgical patients in the MIMIC-IV database, they observed a link between a high anion gap and a higher 90-day all-cause mortality risk in these patients, with the cumulative survival rate being higher in the low anion gap group (20). Concurrently, patients with valvular heart disease, peripheral vascular disease, AKI, and higher AKI stage had a lower predicted 28-day survival rate.

The SOFA score is a valuable tool for predicting short-term mortality in patients with sepsis (1, 21). However, it cannot determine the prognosis of patients with coronary heart disease, as SOFA scores can vary based on the source of infection (22). We evaluated our nomogram by calculating the C-index and AUC, demonstrating that this model performs better than the SOFA score. The clinical benefit of the nomogram, superior to the SOFA score, was further confirmed by calculating the NRI and IDI values. Through DCA, we verified the clinical utility of this nomogram. Our findings indicated that septic patients with hypertension derived more substantial benefits from the nomogram compared to the SOFA score.

Limitations: Our study does have several limitations. Firstly, it was a retrospective analysis using clinical data extracted from the MIMIC-III database, and it has not been validated using other databases or clinical studies. Secondly, the sample size was relatively small, which may have resulted in the exclusion of some potentially significant indicators. Lastly, although the C-index was greater than 0.5, it was less than 0.7 (0.667 and 0.661), indicating room for improvement. These findings guide the direction for future research.

## 5 Conclusion

We developed a nomogram that utilizes age, DBP, pH, lactic acid levels, RDW, anion gap, valvular heart disease, peripheral vascular disease, and AKI grade as indicators to predict the 28-day mortality in septic patients with a history of CHD. This clinical prognosis model is more applicable than the SOFA score for the Sepsis population with CHD.

## Data Availability

The raw data supporting the conclusions of this article will be made available by the authors, without undue reservation.

## Glossary

AKI: Acute kidney injury
ALT: Alanine aminotransferase
APSIII: Acute Physiology Score III
AST: Aspartate amino transferase
AUC: Area under the curve
bpm: Beats per minute
BUN: Blood urea nitrogen
CCU: Cardiac care unit
CHD: Coronary heart disease
CI: Confidence intervals
CRRT: Continuous Renal Replacement Therapy
DBP: Diastolic blood pressure
DCA: Decision curve analysis
HR: Hazard ratio
ICU: Critical care unit
ICD: International classification of diseases
IDI: Integrated discrimination improvement index
INR: International normalized ratio
IQR: Inter-quartile range
LDH: Lactate dehydrogenase
LOS: Lengths of stay
MAP: Mean arterial pressure
MICU: Medical critical care unit
MIMIC: Medical Information Mart for Intensive Care
MIT: Massachusetts Institute of Technology
NRI: Net Reclassification Index
PT: Prothrombin time
PTT: Partial thromboplastin time
Q1: 1st quartile
Q3: 3st quartile
RDW: Red blood cell volume distribution width
SBP: Systolic blood pressure
SD: Standard deviation
SICU: Surgical critical care unit
SOFA: Sequential Organ Failure Assessment
SpO_2_: peripheral oxygen saturation
SQL: Structured Query Language
WBC: White blood cell
Z: Wilcoxon rank sum test
χ^2^: Chi-square test
